# Intricate Plasma-Scattered Images and Spectra of Focused Femtosecond Laser Pulses

**DOI:** 10.1038/srep32056

**Published:** 2016-08-30

**Authors:** C. H. Raymond Ooi, Md. Ridzuan Talib

**Affiliations:** 1 Department of Physics, University of Malaya, 50603 Kuala Lumpur, Malaysia

## Abstract

We report on some interesting phenomena in the focusing and scattering of femtosecond laser pulses in free space that provide insights on intense laser plasma interactions. The scattered image in the far field is analyzed and the connection with the observed structure of the plasma at the focus is discussed. We explain the physical mechanisms behind the changes in the colorful and intricate image formed by scattering from the plasma for different compressions, as well as orientations of plano-convex lens. The laser power does not show significant effect on the images. The pulse repetition rate above 500 Hz can affect the image through slow dynamics The spectrum of each color in the image shows oscillatory peaks due to interference of delayed pulse that correlate with the plasma length. Spectral lines of atomic species are identified and new peaks are observed through the white light emitted by the plasma spot. We find that an Ar gas jet can brighten the white light of the plasma spot and produce high resolution spectral peaks. The intricate image is found to be extremely sensitive and this is useful for applications in sensing microscale objects.

Femtosecond laser pulses amplified through chirp pulse amplification (CPA) technique in Ti:Sa system has sufficient power and intensity to ionize atomic and molecular gases through multiphoton and tunnelling ionization processes, despite its fixed central wavelength *λ* = 800 nm at near infrared region. When passing through a lens, the pulses produce plasma and white light around the focus. We may think that femtosecond laser pulses focused into the air is trivial and unattractive due to the simple setup. Actually there are rich physical processes taking place due to an extremely high electric field. Multiphoton or tunnelling ionization (or both) of nitrogen and oxygen atoms takes place, leading to the creation of a plasma core and a conical section with multiple colors^1^.

Several groups have studied this phenomena over the years. Using femtosecond laser S L Chin found spectral shift and profile that depend on the focusing length of a lens and propagation distance^2^. The same group found that weak/long focusing can create a weak plasma column while tight focusing creates a strong plasma region and suppression of filamentation^3^. Breakup of femtosecond pulse and self focusing into a very narrow beam have been found^4^. Recently, similar results were reported on the breakup of the pulse into two conical sections by slightly tilting the lens with the corresponding spectra that depend on the position of the pulses diffracted by the focused spot^5^. The self-focusing ultrafast Ti: Sapphire laser pulses in air and gas can also produce plasma column composed of highly excited ions, thus providing useful photoemission spectra of atomic and molecular species^6^. High harmonic generation (HHG)^7^ with a broad range of frequencies extending beyond the optical range can occur for laser intensity higher than 10^14^ W/cm^2^.

Here, we study the spectra of white light generated from a focused femtosecond laser pulses into air. The underlying mechanism of a single pulse may be different from laser induced breakdown spectroscopy (LIBS)^8^ but perhaps not the train of pulses with 1 kHz. The laser-plasma interaction process provides a potentially important spectroscopic method where a plasma column is created at the focal spot in a gas by direct photoionization, thus producing spectra with smaller line broadening and a low level of continuum. We show some interesting results in the spectra which enable us to identify the molecular species in the air. By passing through an Ar gas jet, we study the changes in the spectral peaks and the presence of new compounds interacting with Argon gas.

## Experimental Setup

**Figure 1:**
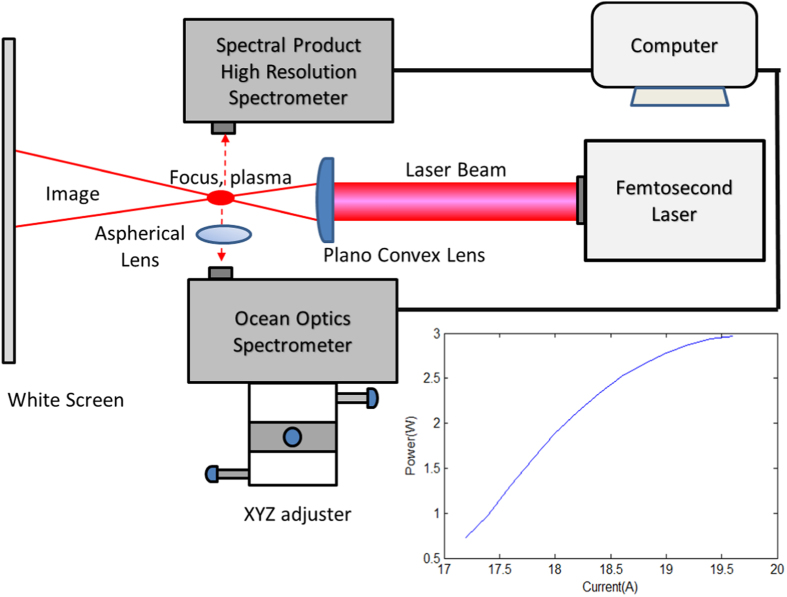
Setup of the experiment with femtosecond laser pulses focused by a plano-convex lens with image on the screen and the white light from the plasma focus being focused into one of the spectrometers. Also shown is the average laser power versus control current plot.

As depicted in Fig. 1, we use Ocean Optics spectrometer for the range 400 nm–600 nm and Spectral Products high resolution spectrometer for 600 nm–1200 nm. Thus we obtain a broad spectrum of the white light in the complementary ranges, along with strong characteristic peaks that can be clearly identified. For our Ti:Sa (Coherent) femtosecond laser system with linear polarization, the pulse duration is *τ* = 48 fs with maximum repetition rate *r* = 1 *kHz* and beam diameter of 1 cm. The laser beam is focused by a plano-convex lens to a spot 12.5 cm away in air at room temperature. The focusing generates a glowing plasma spot that scatters the femtosecond laser beam in the far field. The light from the plasma spot is focused by a 5 mm diameter aspherical lens directly into the spectrometers. For current-power calibration purpose, the power of the laser is adjusted by varying the current on the computer controlled (Evolution) amplifier and the output is measured using a power meter. In experiment, instead of varying the pump energy (that will change the beam quality of the laser) we use a combination of a half-wave plate (hwp) and a polarizer to tune the power output when necessary.

## Laser Parameters

The spatial FWHM of the pulse electric field is defined as 
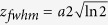
 is related to the pulsewidth *a* along z-direction satisfying the Gaussian profile 
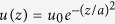
 for energy density.

Assuming a pulse with FWHM *w* the pulse energy *U* can be expressed as


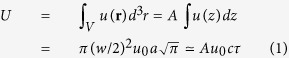


where *u*_0_ (J/cm^3^) 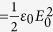 is energy density, *A* = *π*(*w*/2)^2^ is the beam cross section and *τ* = *z*_*fwhm*_/*c* is the temporal FWHM. Hence we can write expressions for the peak power 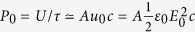, average power *P*_*avg*_ = *rU*, peak intensity *I*_0_(W/cm^2^)
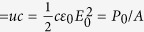
 and peak electric field 
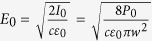
.

Based on the above parameters and the measured average power of *P*_*avg*_ = 3.5 W and the energy per pulse of *U* = 35 mJ, the corresponding to peak power is *P*_0_ = 7 × 10^10 ^W, which is beyond the self-focusing value of 3.77*λ*^2^/8*πn*_0_*n*_2_ ≈ 2 × 10^−9 ^W (*n*_2_ = 5 × 10^−19^ cm^2^/W). Although self-focusing can in principle acts as a lens to produce higher intensity, the creation of plasma channel or even the reversal of Kerr sign^9^ leads to clamping of the intensity at around 10^3^ W/cm^2^ through its defocusing effect. For focused beam, the intensity may be different (higher) than the clamping intensity of 10^13^ W/cm^2^ 10. The actual values of the intensity and the area at the focus are complicated and require exact full simulation of the propagation equation interacting with plasma.

If we may infer the diameter of the focused beam waist from the measurable diameter in laser ablation of Al foil, i.e. *w* = 20 *μ*m, the estimated peak intensity would be in the order of *I*_0_ = 2.3 × 10^16 ^W/cm^2^, corresponding to the peak electric field of 4 × 10^9^ W/cm. The ponderomotive energy is *U*_*p*_ = *λ*^2^*e*^2^*E*^2^/4*m*(2*πc*)^2^ = 2 × 10^−16^ *J*. Taking 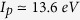 we note that the Keldysh parameter, 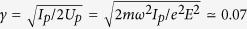 is in the tunnel ionization (TI) regime.

Note that, in vacuum where there is neither plasma defocusing nor Kerr self-focusing the actual focusing spot calculated using the ABCD law is 
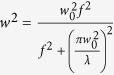
. For *w*_0_ = 1 cm, the focused width turns out to be 3 micron, yielding the intensity much higher than 10^16^ W/cm^2^. However, in reality, there are aberrations that make the focus spot much larger even though plano-convex lens were used.

## Results and Discussions

When the femtosecond laser beam with high compression is focused by a lens, narrow plasma channel is produced in the air at the focus, which scatters an intricate and colorful image on the screen. This is accompanied by a loud high pitch sound at the plasma due to shock wave as atoms/molecules are violently ripped into atoms/ions. The sound diminishes with lower compression.

### Compression

**Figure 2:**
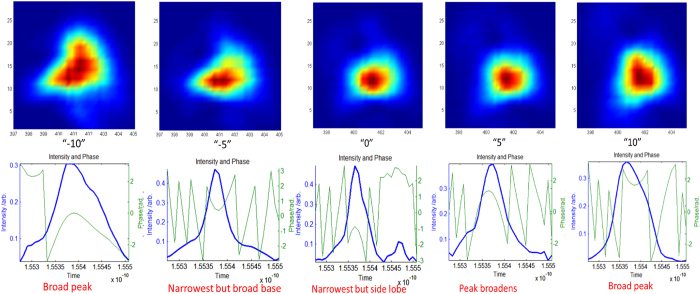
Effects of CPA compression on the FROG spectrogram of femtosecond laser pulses for control current 19A and pulse rate of 1 *k*Hz. The compression is changed by adjusting the delayed (V-shape) mirror in the CPA.

The pulse compression has significant effect on the scattered image, the geometry of the plasma channel and closely connected to the Frequency Resolved Optical Gating (FROG) spectrogram. The FROG spectrograms of the laser pulses are taken for characterization (before focusing) for different compressions, Fig. 2. The highest compression, designated as “0”, corresponds to the intensity of the peak that has the highest value at maximum and is narrowest in time, about 47 fs. The corresponding spectrogram shows a quite rounded and symmetrical distribution over wavelength and delay between the FROG stages.

**Figure 3:**
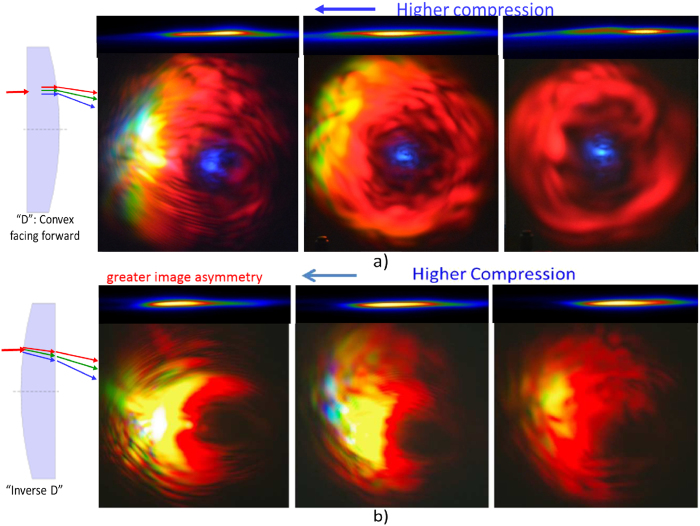
Effects of CPA compression on far field images of femtosecond laser pulses and the corresponding structures of plasma at the focus for two plano-convex lens orientations: the curved lens surface facing (**a**) *forward* and (**b**) *backward*. The control current is 19A and pulse rate is 1 *k*Hz. The ray trajectories for the two lens configurations are shown on the left side.

Figure 3 shows the scattered images at 60 cm from the focused spot. The colors in the scattered image are due to the presence of very strong electric field that enables highly nonlinear optical interactions of the incident femtosecond pulses with the plasma channel, producing shorter wavelength lights over the entire optical domain. This happens only when the compression of the chirp pulse amplification (CPA) is sufficiently high. The compression is controlled by a knob attached to the laser which varies the distance between the compressing mirrors. As the compression drops, the high frequency colors disappear and the image is dominated by reddish color due to scattering of the 800 nm source by the weaker plasma. If an A4 white paper were used as the screen, we observe a **bluish center** due to fluorescence after multiphoton absorption. We do not observe the bluish color on other screens. The scattered images are closely correlated to the plasma, as shown in Fig. 3 for different compression and for two different orientations of the plano-convex lens. The scattered images can be explained by the scattering formula with the source driven by polarization current 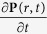 and plasma current **J **= *σ***E**. The theory will be presented elsewhere.

### Plasma structure

The plasma structure is correlated to the intricate and colorful pattern with varying resolution. The scattered image is sensitive to the plasma profile that acts as a scattering medium. Figure 3 shows the corresponding change in the plasma size and intensity with the change in the scattered images as the compression is changed. The plasma is nearly a single highly elongated elliptical shape or needle-like profile without clear structure for both lens configurations: a) “D”- beam from flat surface to convex surface. b) “inverse D”- beam from convex surface to flat surface. There is no single point of focus position due to group velocity dispersion (GVD) of the ultrashort pulse passing through the plano convex lens (in addition to lens aberration). The GVD of the laser pulses is causing the observed cylindrical plasma to have a finite length, the shortest at highest compression for both lens configurations. Although the plasma profile in both configurations look quite identical the scattered images are noticeable different, implying that there are unobserved details and processes within the plasma (like nonlinear wave mixing and phase matching) that determine the intricate pattern of the image. Thus, the intricate image pattern is the result of: a) nonlinear wave mixing that gives the multiple colors and b) scattering by plasma filaments gives the irregular intensity distributions on the image.

The locations of the brightest plasma regions change along z-direction (slightly longer focus) when the compression is reduced. The plasma structure and the scattered image are very sensitive to the lens position and angle relative to the incoming beam. Thus, the plasma structure can be controlled.

**Figure 4:**
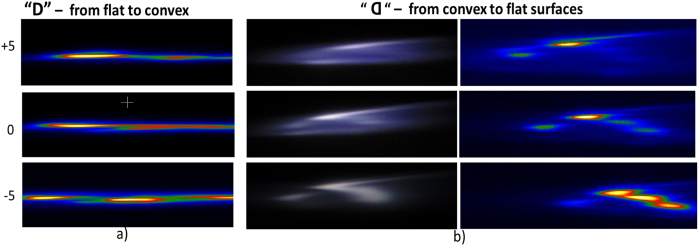
Plasma distributions for two plano-convex lens orientations: the curved lens surface facing: (**a**) *forward* (“D” configuration) and (**b**) *backward*(inverse “D” configuration). The control current is 19A and pulse rate is 1 *k*Hz. The maximum compression is labeled as “0”. The white images of plasma are original color and were taken using normal digital camera.

For the lens with “D” orientation (beam from flat to convex), by tilting the lens slightly, we controllably produce sections of broken plasma aligned almost in one line and a very thin and long channel of plasma within a narrow channel along the optical axis, as shown in Fig. 4a. This should not be confused with or attributed to the refocusing mechanism^11^. The plasma sections merge into one single plasma section with high integrity when the plane of the lens is exactly orthogonal to the beam direction. It shows that the plasma breakup is due to asymmetry during the focusing process.

However, for the “inverse D” orientation (beam from convex to flat), the plasma channel is broader and filled by low density plasma, which contains several distributed high density (multi-filament) plasma not in one line. When the focusing cross section is larger than the D case, the intensity is smaller and the blue shift is smaller (lack of blue light). The intense narrow plasma strip developing on one side, i.e. is the same side of the image with high intensity, see Fig. 4b, contributes to the greater asymmetry in the scattered image (see Fig. 3b).

### Diffraction and Lamellar

When the compression is highest at high power and the beam is directed to the center of the lens at right angle, Fig. 3 shows the fine lamellar structures becomes very clear and the sound of the shock becomes very loud. The high laser intensity creates (high integrity) dense plasma channel which acts as a nearly cylindrical barrier to the laser pulses which expels the beam away from it and creating the clearer diffraction pattern^10^ as the lamellar structure. Based on the diffraction formula, and 0.1 degree between lamellar lines, we estimate the plasma diameter to be 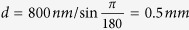, in agreement with the observation. with the corresponding narrow thin needle channel of plasma which produces the intricate lamellar structure through diffraction. As the compression is reduced, the integrity of the plasma spot is reduced, hence the diffraction effect is lost. As the compression is reduced, the plasma spot becomes dimmer and this causes the disappearance of the fine lamellar pattern and the image is essentially reddish. This implies that the **integrity** of the plasma channel is important for scattering the laser pulses to produce image with high resolution lamellar structures.

### Colorful image

At high power, control current above 17.3A, the emission cone contains region of yellow, green and blue/purple patches with **shorter wavelengths** at some distances from the center of the spot. They are due to self-phase modulation, upconversion and second (and even third) harmonics from the highly nonlinear process resulting in multiple filamentations. This effect is opposite to the normal beam diffraction where color is red-shifted away from the center, according to the diffraction formula *d* sin *θ* = *mλ*. Nonlinear frequency conversion produces the greenish and yellowish regions at the edge of the image. For D configuration, the peak electric field or intensity is higher than the inverse D case, thus generating lights in the blue region. As the compression is reduced, the greenish and yellow regions disappear as the plasma spot becomes less intense.

### Asymmetry

The pattern on the images Fig. 3 appears asymmetric because of small asymmetry in the beam that is being amplified by the lens. The asymmetry is more acute when the beam is slightly off-centered from the lens, and the colored images become brighter. When the planar-convex lens is rotated 180 degrees such that the flat surface of the lens is at the backside while the convex surface facing the forward direction (case “D”), the **asymmetry** of the image is reduced, as seen in Fig. 3a. The images are more symmetric as the path of a ray is refracted only once (at the 2nd interface that is curved) compared to the other case, as illustrated in Fig. 3b. This is because any small spatial asymmetry would be amplified more in the case of the beam going from convex to flat surfaces where it takes a longer path, has greater dispersion, spherical and chromatic aberrations as well as undergoing 2 times refraction (change of ray directions).

### Spectra of Colors

**Figure 5:**
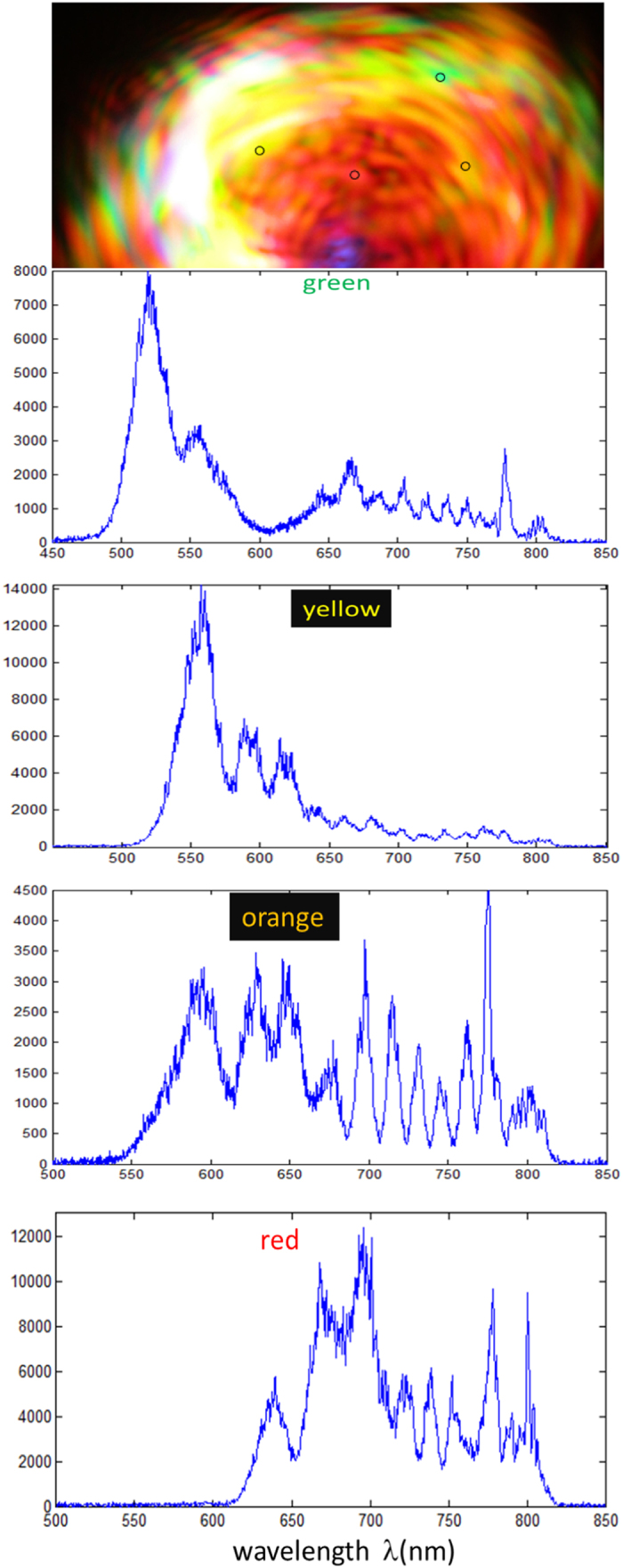
Spectra of colored spots on the scattered image. Small circles show green, yellow, orange and red colored spots on the image of scattered femtosecond laser pulses (average power 3 W) after scattering by the white plasma at the focus.

Figure 5 shows the spectra for green, yellow, orange and red spots on the scattered image. Notice that the spectra of each color is composed of a broad range of blue-shifted frequencies with the peaks produced by self-phase modulation (SPM) through the nonlinear refractive index or Kerr effect *n*_2_*I* as well as plasma dispersion. We propose a simple and effective explanation for the oscillations as due to the interference between the scattered pulse with the incident pulse. The presence of fractions of delayed pulses after focusing has been verified in the FROG spectrogram. The scattered pulse is frequency **blue-shifted** and delayed by time *t*_*p*_ = *L*_*p*_/*c* due to the plasma of effective length *L*_*p*_, typically less than 3 mm and depending on nonlinearly scattered frequency. It is known that the Fourier transforms of two pulses separated by *t*_*p*_ in time would give oscillations with frequency


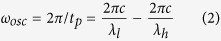


corresponding to the separation between peaks of higher(*λ*_*h*_) and lower(*λ*_*l*_) wavelengths of


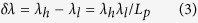


For plasma length of *L*_*p*_ ≈ 3 *mm* we have *δλ* = 17 *nm* which agrees with the peak separation in Fig. 5 around 700 nm. The lower wavelengths photons are scattered at a wider angle, thus the effective plasma length would be slightly smaller, hence the separation between the peaks is larger. Energy is compromised between the blue-shifted photons (which have higher energy) from nonlinear conversion and plasma generation energy through photoionization. Thus, more blue-shifted photons corresponds to plasma with lower intensity, in agreement with Fig. 3.

### Rate

**Figure 6:**
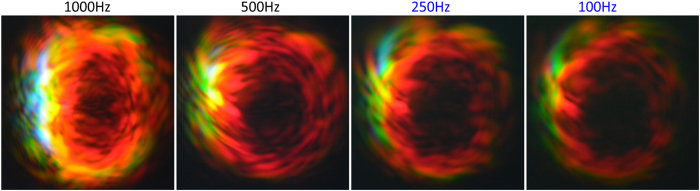
Effects of pulse repetition rate on far field images of femtosecond laser pulses after scattering by the focused white light spot. The control current is 19A with curved surface of lens facing *backward* direction.

The repetition rate has little effect on the features of the scattered image for rate of 250 Hz and below, as shown in Fig. 6 for current 19.0 A. The intricate lamellar or wavy pattern (due to diffraction of the plasma spot) is not so clearly seen below 500 Hz. However, for rate 500 Hz and above, the image is changed noticeably. Blue and violet patches (of higher frequencies) begin to appear and the intricate lamellar interference pattern becomes quite prominent. This is due to the scattering by high integrity/density plasma generated by high field intensity. This implies that the integrity of the plasma is affected by the repetition rate above 500 Hz. The lifetime of plasma generated by a single femtosecond laser pulse is less than 100 ns^12^. At 500 Hz or 1 kHz rate, the plasma lifetime (much shorter than the duration between two pulses) should not be affected by the multiple pulse effect. Therefore, each pulse must have left some residual effect on the ambient gas or plasma lasting through few milliseconds such that the next pulse interaction would be affected by some effect of the previous pulse, giving rise to multi-pulse interactions. This is not entirely unexpected as it has been shown that with a sequence of 17 pulses separated by 30 ns apart, the plasma lifetime can be prolonged to 1 *μs*^12^, in analogy to the quantum anti-Zeno effect.

We can conclude that the effect of the pulse repetition rate on the intricate image must involve slower dynamics, particularly the residual plasma that lasts till a few milliseconds, and possibly thermal heating of the plasma, that affect (enhance) the integrity of the plasma. We have learned that clear and bright (or high integrity) plasma would produce clear lamellar structure on the image. On the other hand, the loss of lamellar is due to weakening of the plasma when the rate reduces, i.e. the clarity of the fine lamellar structure reduces.

**Figure 7:**
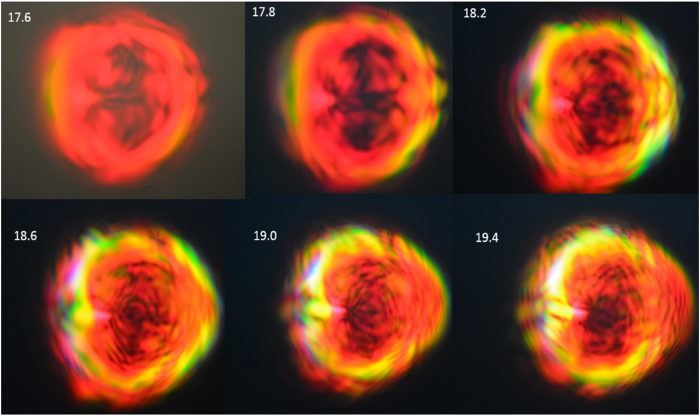
Effects of different powers (control current 17.2A…19.4A) on far field images of femtosecond laser pulses after scattering by the focused white light spot for 1 *k*Hz rate with curved lens surface facing *backward*.

We also capture series of images for different power levels, as shown in Fig. 7 for different powers which show that the increase in the current above 18A does not affect the intricate structure of the scattered image as long as the intensity already exceed the level where nonlinear processes can already occur to produce the intricate image structures.

### Spectroscopy of plasma

**Figure 8:**
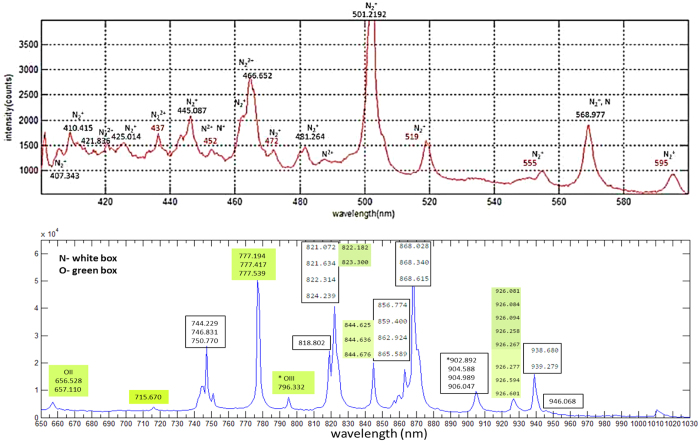
Spectra of the white light due to focused femtosecond laser pulses in the ranges 400 nm–600 nm (with Ocean Optics HR4000) and 600 nm–1200 nm (Spectra- Physics spectrometer) with an integration time of 5 ms. The mean laser power here is 3 W with repetition rate of 1 kHz focused by a 12.5 cm plano-convex lens, as illustrated in Fig. 1.

**Figure 9:**
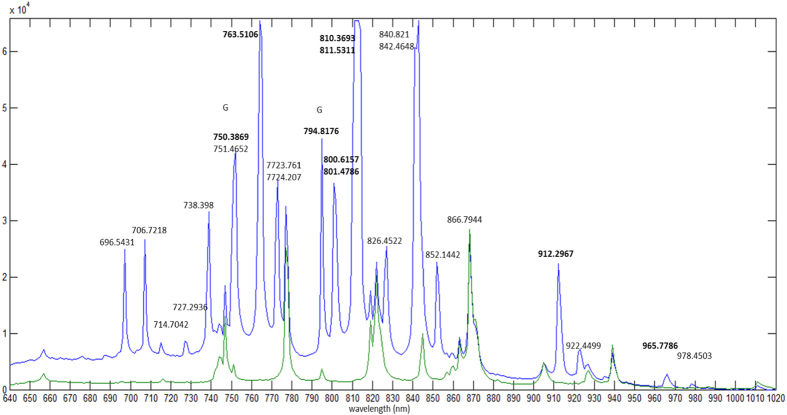
Spectra of the white light from focused femtosecond laser pulses on the Argon gas jet (red) compared with air (blue). The bold numbers indicate strong lines while G reminds that the peak for Ar almost coincides with the peak for N.

The spectra in Figs 8 and 9 correspond to the white light of the focused plasma spot that were taken perpendicular to the laser beam axis while the colored pictures in previous figures are images scattered by the plasma taken in the forward direction. The white light at the focus is due to violent kinetics of electrons being stripped off to form a plasma through quantum tunnelling and recollision of energetic and accelerated electrons with the ions emitting harmonics over the entire optical region that is seen as a white light that can be used as a probing technique for molecules in the air^13^ and combustible gaseous^14^,^15^. The main peaks in the spectra Fig. 8 are due to atomic nitrogen, ionized atomic nitrogen and ionic molecular nitrogen being ionized 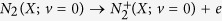 or ionized and being excited to *B* through 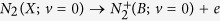. Subsequent decays produce the emission spectra.

**Figure 10:**
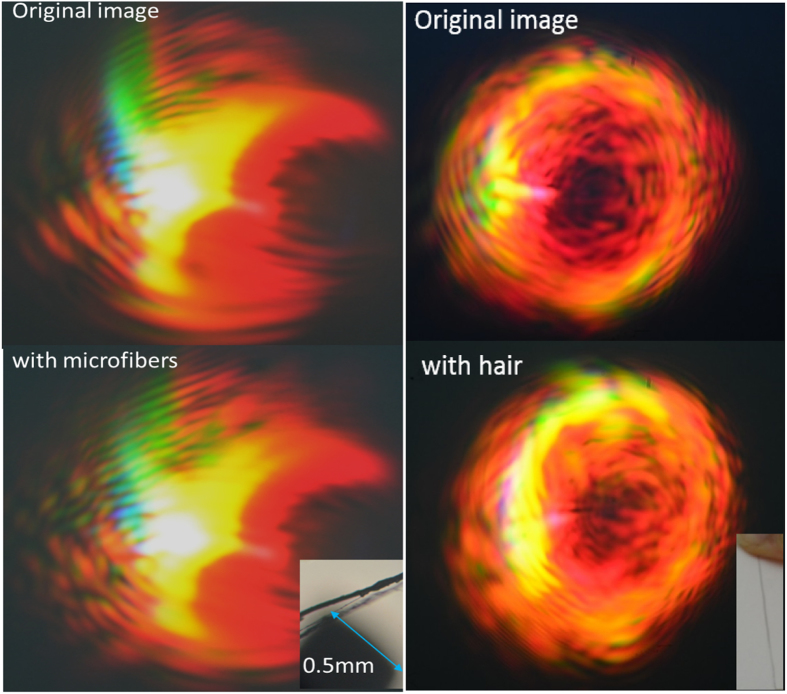
Microscope image of dust web microfibers near the tip of a caliper. The original image is altered by the microfibers introduced just before the pulses impinge the plano-convex lens.

Figure 10 we cannot determine Fig. 8 (lower panel) only shows the presence of neutral atomic *N* and not singly ionized atomic nitrogen *N*^+^ based on refs 16 and 17 since their spectral peaks are in VUV with wavelengths 10 times shorter than the detectable range of our spectrometers. From the data in ref. 18 we obtain good match of the peaks for atomic *N* and singly ionized atomic nitrogen *N*^+^ with the spectrum Fig. 8. This shows the presence of atomic *N* and *N*^+^ as the breakdown products of the intense laser fields.

The numbers for the characteristic peaks in Fig. 8 are obtained from NIST data^19^ for the strongest lines for atomic N and O and they also agree with those from ref. 18. The main peaks of the spectrum in Fig. 8a in the range 400 nm–520 nm coincide with the main peaks of Fig. 3 in ref. 6. In the range 600 nm–1000 nm, all the peaks in the spectrum of Fig. 8b are clearly identified, and they reproduces that of Fig. 2c in ref. 3 but with much better resolution. In addition, we observe new lines (*OIII at 796.332 nm and O1 around 902–906 nm) that were predicted (Ritz) but were never observed according to the NIST data.

The resolution of the spectra obtained in ref. 3 were not so high, only strong lines identified were atomic lines *O* 777.4 nm, 822.7 nm and *N* 746.6 nm, 868.6 nm^20^. The strong lines were recently being used for lasing^21^. Here, the lines of neutral *N*_2_ are most dominant^18^ while the lines for singly ionized 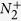 are hardly visible. At higher frequency range, the peaks show the presence of 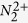 in agreement with the similar experiment^6^ but that was done for wavelengths below 650 nm only. Strong lines of molecular oxygen ions 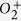 and lines due to neutral or ionized *O*_2_^22^ are hardly observed within the range.

We now inject a jet of Ar gas through the plasma focus. We find additional peaks of Ar appearing, in addition to the peaks for ambient air (Fig. 9). The spectroscopic studies of mixture of *N*_2_ and Ar is useful and of interest for fluorescence lighting^23^. The white light spot of the plasma at focus glows brightly. The intensity of the plasma increases with the jet pressure. New peaks emerge within 600–1000 nm are due to Ar I (ground state atomic Ar)^24^. Peaks for Ar II (Ar^+^ ions)^25^ kare not observed within this range because they fall within the VUV region^26^. In addition, it seems that there are amplified peaks of *N*_2_, but actually they are due to Ar I lines^19^ that almost coincide with the atomic *N* lines.

### Sensitive Detection of Micro-objects

We notice in Fig. 10 that the image is very sensitive to any tiny object present in the path of the laser beam. Even a fiber (from dust web) with dimension as small as 10 micrometer introduced just behind the plano-convex lens can cause a contrasting change to the delicate lamellar pattern. The original image serves as a standard fingerprint of the background and a tiny foreign object would distort the delicate interference pattern to a noticeable level, as seen by comparing the images in Fig. 10. Thus we predict that this effect has an application for sensing of tiny objects and even small movement of the air can be detected through image processing. A simple setup with fiber-based femtosecond laser and lenses may be used in a high security alarm facility to detect any intrusion.

In conclusion, optical image scattered by focused plasma has rich intricate pattern that depends on the pulse parameters such as power, repetition rate and CPA compression. We explain how the orientations of the plano-convex lens lead to distinct scattered images. While the plasma provides the spectroscopic information of the ionic and molecular constituents of the background, the shape, diameter and density of the plasma channel are connected to the detailed features of the scattered image, including the lamellar structure due to diffraction of the plasma. The structure of the plasma can be controlled and can also explain spectral oscillations of colored spots in the image due to time delay of interfering pulses. We show that the intricate pattern in the image is extremely sensitive to smaller perturbation and can be an important asset for sensing the presence of tiny microscopic objects.

## Additional Information

**How to cite this article**: Ooi, C. H. R. and Ridzuan, M. Intricate Plasma-Scattered Images and Spectra of Focused Femtosecond Laser Pulses. *Sci. Rep.* **6**, 32056; doi: 10.1038/srep32056 (2016).
